# Propagation Structure of Intrinsic Brain Activity in Migraine without Aura

**DOI:** 10.3390/brainsci12070903

**Published:** 2022-07-10

**Authors:** Lingling Dai, Qiang Xu, Xing Xiong, Yang Yu, Ximing Wang, Hui Dai, Hongru Zhao, Jun Ke

**Affiliations:** 1 Department of Radiology, The First Affiliated Hospital of Soochow University, Suzhou 215000, China; fmridll@126.com (L.D.); sudaxiongxing@163.com (X.X.); yuyang921502@163.com (Y.Y.); wangximing1998@163.com (X.W.); huizi198208@126.com (H.D.); 2 Institute of Medical Imaging, Soochow University, Suzhou 215000, China; 3 Department of Medical Imaging, Jinling Hospital, Medical School of Nanjing University, Nanjing 210002, China; xuqiangnj12@163.com; 4 Department of Neurology, The First Affiliated Hospital of Soochow University, Suzhou 215000, China

**Keywords:** migraine, dynamics, resting-state, functional magnetic resonance imaging

## Abstract

Previous studies have revealed highly reproducible patterns of temporally lagged brain activity in healthy human adults. However, it is unknown whether temporal organization of intrinsic activity is altered in migraines or if it relates to migraine chronification. In this resting-state functional magnetic resonance imaging study, temporal features of intrinsic activity were investigated using resting-state lag analysis, and 39 episodic migraine patients, 17 chronic migraine patients, and 35 healthy controls were assessed. Temporally earlier intrinsic activity in the hippocampal complex was revealed in the chronic migraine group relative to the other two groups. We also found earlier intrinsic activity in the medial prefrontal cortex in chronic compared with episodic migraines. Both migraine groups showed earlier intrinsic activity in the lateral temporal cortex and sensorimotor cortex compared with the healthy control group. Across all patients, headache frequency negatively correlated with temporal lag of the medial prefrontal cortex and hippocampal complex. Disrupted propagation of intrinsic activity in regions involved in sensory, cognitive and affective processing of pain may contribute to abnormal brain function during migraines. Decreased time latency in the lateral temporal cortex and sensorimotor cortex may be common manifestations in episodic and chronic migraines. The temporal features of the medial prefrontal cortex and hippocampal complex were associated with migraine chronification.

## 1. Introduction

Migraines are a common neurologic disorder, with the 1-year prevalence estimated to be around 15.3% in the general population (18.1% in women and 9.1% in men) [1]. The typical manifestations are attacks of recurrent, unilateral, moderate to severe, throbbing and pulsating headaches that are frequently aggravated by movement and associated with nausea, vomiting, and sensory hypersensitivity [2]. Migraines could affect occupational, academic, social functioning of patients, leading to low individual quality of life and high economic burden for the society. According to the 2019 Global Burden of Disease study, migraines are the second leading cause of disability worldwide, but first among young women [3]. However, the pathogenesis of migraines is complex and, thus far, remains incompletely understood.

In the past few decades, advanced neuroimaging modalities have been increasingly used in the uncovering of migraine pathophysiology. It is now widely accepted that migraines are best understood as a neuronal network disorder [4]. Resting-state functional MRI is an imaging technique with great promise in examining coordinated activity between different brain regions, in the absence of any stimulation or tasks [5]. Utilizing this approach, multiple brain structures involved in the processing of pain, such as the hypothalamus, periaqueductal gray matter, hippocampus, amygdala, insula, basal ganglia, thalamus, sensorimotor and visual cortex, have been revealed to present atypical functional connectivity (FC) in migraine patients during the interictal phase [6,7,8,9]. Furthermore, several imaging studies on migraines have reported disrupted connectivity within or between resting-state networks, including the salience network, frontoparietal network, and default mode network (DMN) [6,7].

It is worth noting that resting-state FC findings across migraine studies are often inconsistent and even conflicting. The variations in results between studies may be partially attributed to clinical heterogeneity of migraine patients (migraine types, presence of aura) and methodological difference in data analysis [6,10,11]. For example, while some investigators used a seed-based correlation approach, others utilized a data-driven method, such as spatial independent component analysis. In order to facilitate comparative assessment, it is proposed that whole-brain or data-driven analyses, rather than analyses based on region of interest, should be more extensively implemented [7,12]. Notably, because synchronization of spontaneous brain activity is widely referred to as FC, implicit in conventional resting-state FC analysis is the assumption that intrinsic brain activity is exactly temporally synchronous between functionally related regions. However, several lines of evidence have indicated that the intrinsic brain activity is spatiotemporally structured in humans and mice [13,14,15]. Unfortunately, most functional MRI studies on resting-state FC have overlooked the temporal feature of intrinsic brain activity.

Mitra et al. specifically focused on this issue and found reproducible temporally lagged patterns of spontaneous brain activity in normal humans in a resting-state functional MRI study [16]. Furthermore, the temporal latency structure was revealed to change with experiment manipulation of the behavior state, suggesting that the lags in intrinsic activity represent neuronal processes rather than hemodynamic delay [16]. Taken together, it is reasonable to speculate that temporal lag analysis of spontaneous brain activity may provide new insights into the brain function of humans during disease states. Indeed, altered propagation of intrinsic brain activity has been detected in several neuropsychological conditions, including autism [17], epilepsy [18] and posttraumatic stress disorder [19]. However, it is unknown whether the lag structure was disrupted in migraines or is related to the subtypes of this disorder.

Therefore, the current study aimed to explore the impact of migraines on the temporal structure of intrinsic brain activity by preforming a time lag analysis of resting-state functional MRI data. Temporal lag differences between patients with episodic migraines (EM) and chronic migraines (CM) were compared to investigate whether this measure varied in patients with distinct headache frequency. Based on the existing findings on migraines, we hypothesized the temporal lag structure of migraine patients may be altered in regions participating in different domain of pain processing, including the hippocampus and prefrontal cortex. Moreover, the EM group and the CM group may exhibit different temporal lag structures, and the time latency difference may be associated with clinical indices reflecting disease severity, such as headache frequency.

## 2. Materials and Methods

### 2.1. Participants and Clinical Assessment

The ethics committee of the first affiliated hospital of Soochow University approved this study (Approval no. 2021246), which was performed in accordance with the Declaration of Helsinki. Written informed consents were acquired from all subjects prior to their participation. A total of 56 migraine patients (39 with EM without aura and 17 with CM without aura) and 35 healthy control (HC) subjects were enrolled in the present study. All participants were aged 18–65 years and right-handed. The migraine patients were recruited from the headache outpatient clinic. Migraine diagnosis was established according to the criteria in the International Classification of Headache Disorders (ICHD-3 beta) [2]. The clinical assessment of migraines was carried out by a single expert neurologist. Migraine patients were asked about the accurate clinical history, comprising their headache frequency (average headache days per month) and disease duration (years with migraine). The headache pain intensity was evaluated with a 10-point visual analog scale (VAS). The HC subjects were recruited via advertisement; they should have not suffered from migraines or other types of headaches, and had no family history of migraine. To avoid brain function changes related to acute migraine symptoms, EM patients should be free of migraine attacks in the 72 h before the examination and 48 h after the MRI scans [20]. For CM patients, we managed to perform the imaging assessment during the headache-free interval. The CM patients were considered interictal if they did not experience a headache that led to acute migraine medication usage, in the 24 h before and after the MRI acquisition. In addition, migraine patients were excluded if they had a medication overuse headache or were under any preventive therapy for migraines in the 3 months prior to participating this study. General exclusion criteria for all individuals included presence of current or past psychiatric disorders; other neurological disorders or pain conditions; serious medical disorders, such as cardiovascular and metabolic disease; drug or alcohol abuse; contraindications to MRI; and excessive movement during the MRI scanning (head translation > 1.5 mm or rotation > 1.5° at any direction).

### 2.2. MRI Data Acquisition

Brain imaging was conducted with a 3.0 Tesla scanning system (MAGNETOM Skyra, Siemens Healthcare, Erlangen, Germany) at the Department of Radiology, the first affiliated hospital of Soochow University. To acquire high-resolution T1-weighted anatomic images, a sagittal fast spoiled gradient recalled echo sequence was used with the following parameters: repetition time (TR) = 2300 ms, echo time (TE) = 2.98 ms, matrix = 256 × 256, field of view (FOV) = 256 × 256 mm^2^, slice thickness = 1 mm. The structural images were then examined by two experienced radiologists to exclude the presence of clinically silent lesions. The resting-state functional MRI data were obtained with an axial echo-planar imaging sequence (TR = 2000 ms, TE = 30 ms, flip angle = 90°, FOV = 256 × 256 mm^2^, matrix = 64 × 64, number of slices = 33, slice thickness = 4 mm, no intersection gap, number of volumes = 240). During the functional imaging, all participants were instructed to relax with their eyes closed, remain awake and do not think about anything in particular.

### 2.3. Data Preprocessing

The imaging data preprocessing was accomplished using Statistical Parametric Mapping (SPM12, http://www.fil.ion.ucl.ac.uk/spm/ (accessed on 14 June 2022)) and the Data Processing Assistant for Resting-state Functional MRI advanced edition (DPARSFA, http://www.restfmri.net (accessed on 14 June 2022)). For the functional images, we removed the first 10 volumes of data to reduce the influence of instability magnetization. The following steps included slice-time correction, realignment, and co-registering with the individual high-resolution structural images. The co-registered T1- weighted images were then segmented into gray matter, white matter (WM), and cerebrospinal fluid (CSF), and spatially normalized into the standard Montreal Neurological Institute (MNI) space using a final size of 3 × 3 × 3 mm^3^. We then applied the resulting normalization matrix to the resting-state functional images. Moreover, removal of linear trends and band-pass filtering (0.01–0.08 Hz) were performed to reduce the low-frequency drift and high frequency physiological noise. The obtained images were subsequently spatially smoothed by convolution with an 8-mm full-width half maximum, isotropic Gaussian kernel. To reduce the effects of the confounding factors, nine nuisance covariates, including six head motion parameters, time series predictors for global mean signal, WM signal and CSF signal were regressed from the time series. For additional functional MRI data preprocessing, frame censoring was applied by calculating the DVARS (differentiated rms variance) measure, with a threshold of 0.5% frame-to-frame image intensity change [21,22]. Subjects with less than 150 retained frames for the resting-state functional image were not included in the further analyses. The fraction of removed frames in the functional MRI data was 15.64% ± 22.7%, 20.2% ± 23.9% and 12.8% ± 15%, respectively, in the EM, CM and HC groups. No significant difference in the number of censored frames of data was detected among the three groups. Finally, de-noising was conducted by utilizing a combination of approaches, as described in previous studies [23,24].

### 2.4. Resting-State Lag Analysis

Conventional strategies of FC analysis incorporate the assumption of exact temporal synchrony (zero-lag correlation) for a given time series. By contrast, the current study specifically analyzed the temporal organization of intrinsic activity as expressed in its latency structure. The method for computing lags between time series has been published in prior literature [16]. Accordingly, the lagged cross-covariance functions were evaluated according to the following formula:

(1) $$Cx_{i}x_{j}\left(\tau \right)=\frac{1}{T}\int x_{i}\left(t+\tau \right)\times x_{j}\left(t\right)dt_{}$$

where τ represents the temporal lag (equivalently, time delay, *TD*), and *T* is the interval of integration. The value of τ where $Cx_{i}x_{j}\left(\tau \right)$ displays an extremum defines the temporal lag between time series $x_{i}\left(t\right)$ and $x_{j}\left(t\right)$. The extremum is determined using parabolic interpolation. Due to the aperiodicity of the blood oxygen level-dependent (BOLD) signal, this process almost always yields a single, well-defined extremum, with the corresponding τ typically in the range ±1 s.

For a given set of n time series, {*x*_1_(*t*), *x*_2_(*t*), …, *x_n_*(*t*)}, the $\tau_{ij}$ between each pair of time series corresponding to the extremum of $Cx_{i}x_{j}\left(\tau \right)$ could be computed, resulting in an antisymmetric, *TD* matrix, which is as follows:

(2) $$TD=\left[\begin{array}{ccc} \tau_{1,1} & \cdots & \tau_{1,n}\\ \vdots & \ddots & \vdots \\ -\tau_{1,n} & \cdots & \tau_{n,n} \end{array}\right]$$

The diagonal elements of *TD* are necessarily zero, as any time series has zero lag with itself. Moreover, $\tau_{ij}$ = $-\tau_{ji}$, since a time series $x_{i}\left(t\right)$ preceding $x_{j}\left(t\right)$ indicates that $x_{j}\left(t\right)$ follows $x_{i}\left(t\right)$ with the same interval.

Subsequently, the multivariate data represented in the *TD* matrix were projected onto one-dimensional maps by applying the technique described by Nikolic and colleagues [25,26]. To be specific, we computed the lag projection as the mean of elements in each column of *TD* matrix (Equation (2)), that is,

(3) $$Tp=\left(1/n\right)\left[\overset{n}{\underset{j=1}{\sum }}\tau_{1,j}\cdots \overset{n}{\underset{j=1}{\sum }}\tau_{n,j}\right]\;$$

The voxel-wise Tp indicates whether the BOLD signal for a given voxel is earlier or later than the rest of the brain. Group-level lag projections were then obtained by averaging the voxel-wise Tp maps of each individual for the EM group, CM group and HC group, respectively.

### 2.5. Statistical Analysis

The SPSS 16.0 (SPSS Inc., Chicago, IL, USA) was utilized to test statistical significance of difference in the demographic data and clinical measures, with a significance threshold *p* < 0.05. Chi-squared test was applied to analyze gender distribution difference among the groups. Age and education level were compared by conducting one-way analysis of variance (ANOVA). For migraine-related clinical measures, an independent *t*-test was used to evaluate the differences between the EM and CM groups.

The resting-state lag projection maps were compared among the groups by performing ANOVA with SPM12, using age, gender, and education level as the nuisance covariates. Once significant differences were revealed by ANOVA, post-hoc *t*-tests were conducted to assess the difference between each pair of groups. For ANOVA and post hoc *t*-tests of lag projection maps, the significance threshold was set at *p* < 0.05. The results were multiple comparison corrected based on Gaussian random field theory (voxel-level *p* < 0.01, cluster level *p* < 0.05, two tailed) by using the Data Processing and Analysis for Brain Imaging (DPABI, http://rfmri.org/dpabi (accessed on 14 June 2022)) package.

Furthermore, lag values of brain regions with significant group differences were extracted and correlated against the clinical variables across all migraine patients to explore the relationship between migraine symptoms and temporal lag structure of resting-state brain activity. For this purpose, Pearson correlation analyses were performed with SPSS 16.0 using a significant level of *p* < 0.05 (Bonferroni corrected for multiple comparisons).

## 3. Results

### 3.1. Participants’ Characteristics

Participants’ characteristics are provided in Table 1. There was no significant difference regarding gender proportion (*p* = 0.06) or education level (F = 1.663, *p* = 0.195) among the three groups. However, the mean age of the CM group was found to be higher than that of the other two groups (F = 8.627, *p* < 0.001). Headache frequency in the CM group was significantly higher than that of the EM group (*p* < 0.001). No significant difference in pain intensity, as reflected by VAS, was observed between the two patient groups (*p* = 0.635).

### 3.2. Resting-State Lag Analysis

**Figure 1:**
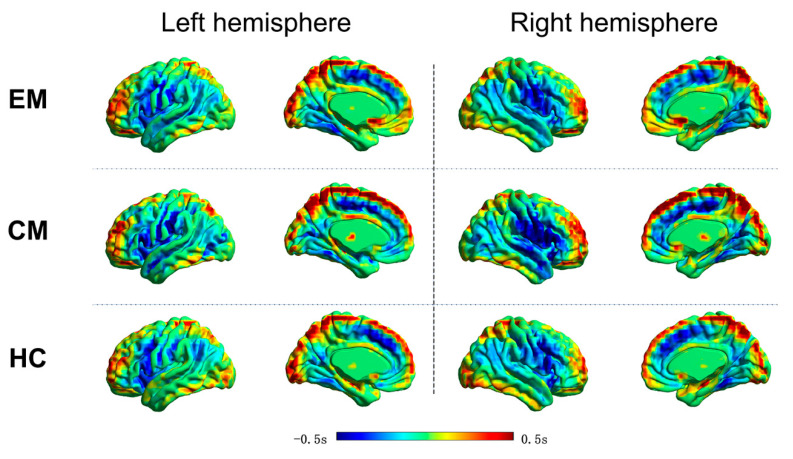
Temporal lag projection maps in the EM, CM, and HC groups. Warm color indicates brain areas in which intrinsic activity is temporally later, relative to that of the rest of the brain (positive seconds). Cool color indicates brain areas in which intrinsic activity is temporally earlier, relative to that of the rest of the brain (negative seconds). Apparent propagation of resting-state brain activity is measured on a time scale of ±0.5 s. EM, episodic migraine; CM, chronic migraine; HC, healthy control.

Figure 1 displays the group-level lag projection maps, which showed whether the BOLD signal of a given voxel was, on average, early (blue) or late (red), with respect to the rest of the brain. In the HC group, the resting-state latency structure was highly bilaterally symmetric, and distinct early and late brain areas were detected. The time lag value mainly ranged from −1 s to 1 s, indicating that intrinsic brain activity propagated through and across networks on a timescale of about 1 s. Brain regions that showed apparently earlier intrinsic brain activity included the posterior cingulate cortex/precuneus and the dorsal anterior cingulate cortex/dorsal medial prefrontal cortex. The EM and CM groups showed similar topography in the lag projection maps to that of the HC group.

**Figure 2:**
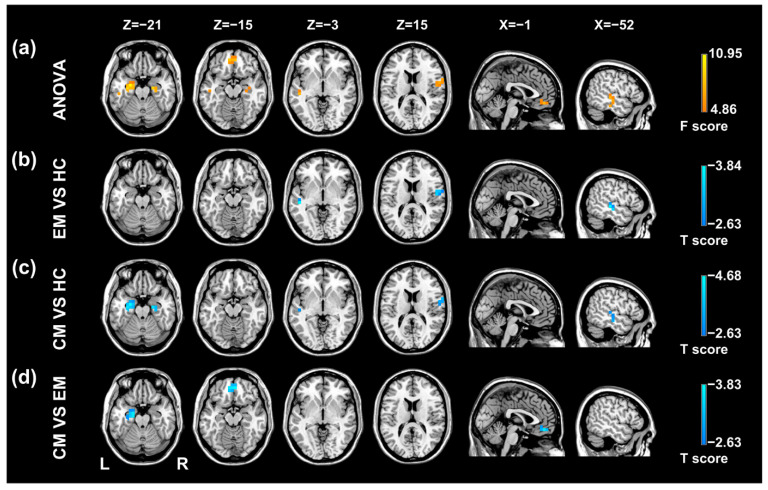
Results of statistical analyses of temporal lag of intrinsic brain activity in the migraine patients and healthy controls. (**a**) Regions showing significant differences in temporal propagation among the three groups include the left STG/MTG, right PoCG/PreCG, bilateral HP/PHG and mPFC. (**b**) Temporal propagation differences between the EM group and HC group. (**c**) Temporal propagation differences between the CM group and HC group. (**d**) Temporal propagation differences between the CM group and EM group. EM, episodic migraine; CM, chronic migraine; HC, healthy control; STG, superior temporal gyrus; MTG, middle temporal gyrus; PoCG, postcentral gyrus; PreCG, precentral gyrus; HP, hippocampus; PHG, parahippocampal gyrus; mPFC, medial prefrontal cortex.

As shown in Table 2 and Figure 2, there were significant lag differences among the groups in the bilateral hippocampus/parahippocampal gyrus (HP/PHG), medial prefrontal cortex (mPFC), right postcentral gyrus/precentral gyrus (PoCG/PreCG), and left superior temporal gyrus/middle temporal gyrus (STG/MTG). Post hoc *t*-tests demonstrated that the BOLD signal of the mPFC was temporally earlier than the rest of the brain in the CM versus EM group comparison (Table 3, Figure 2). The CM group showed earlier intrinsic activity in the bilateral and left HP/PHG, respectively, when compared with the HC group and the EM group (Table 3, Figure 2). BOLD signals of the left STG/MTG and right PoCG/PreCG were temporally earlier, with respect to the rest of the brain in both migraine groups relative to the HC group (Table 3, Figure 2).

### 3.3. Correlation Analyses

**Figure 3:**
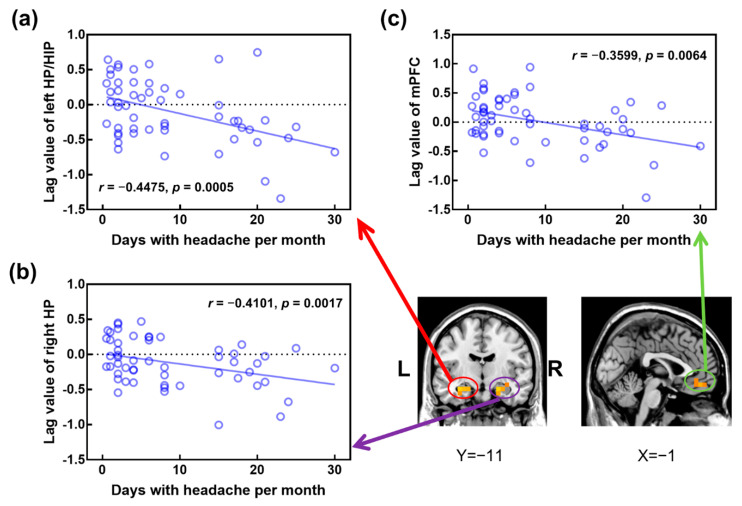
Significant correlation between headache frequency and temporal lag of intrinsic activity. Across all migraine patients, the number of days with headache per month is negatively correlated with temporal lag value in the left HP/PHG (**a**), right HP/PHG (**b**), and bilateral mPFC (**c**). HP, hippocampus; PHG, parahippocampal gyrus; mPFC, medial prefrontal cortex.

Temporal lag values in both the left HP/PHG and right HP/PHG were significantly anticorrelated with headache frequency across all patients with migraines (r = −0.4475, *p* = 0.0005; r = −0.3599, *p* = 0.0064, respectively) (Figure 3a,b). In addition, there was a negative correlation between headache frequency and temporal lag values in the mPFC (r = −0.4101, *p* = 0.0017) (Figure 3c). No statistically significant correlation was revealed between other clinical measures of migraines and regional lag values in patients with migraines.

## 4. Discussion

We applied resting-state lag analysis to investigate the temporal organization of intrinsic brain activity in migraine patients and HC subjects. It has been previously shown that examining the lag structure could provide important information about the abnormal brain function underlying various neurologic and psychiatric disorders [17,19]. Although the mechanisms of propagated intrinsic activity have not been fully elucidated, the balance in excitatory and inhibitory activity [27] and astrocytic signaling [28] may be possible contributors of this phenomenon. Our results revealed abnormal propagation pattern in the left PoCG/PreCG, right STG/MTG and biliteral HP/PHG in patients with migraines. Focal differences in the mPFC and left HP/PHG existed between the CM and EM groups, and correlated with headache frequency across all patients, suggesting that disrupted temporal lags may reflect the disease burden of migraines.

In the present study, the CM patients showed significantly decreased temporal lag values (early intrinsic activity) in the HP/PHG, compared with the EM patients and HC subjects. The temporal lag value of the mPFC was significantly decreased only in the CM versus EM comparison. Specifically, the temporal lag values in the HP/PHG and mPFC were positive or nearly zero in the EM and HC groups, but were strongly negative in the CM group, indicating that these two regions were a stronger source of propagated intrinsic activity in patients with CM. Partially consistent with our result, altered effective connectivity of both the HP/PHG and mPFC has been observed in migraine patients in previous studies [29,30,31]. The HP/PHG plays important roles in learning and memory, stress response [32], as well as pain-related attention and anxiety [33]. Migraines itself could be considered as a stressful event [34]. Thus, increased intrinsic activity propagation from HP/PHG in migraine patients identified in the current study may reflect maladaptive stress responses and enhanced attention to pain. The mPFC is involved in pain modulation via cognitive mechanisms [35]. We speculative that our finding of increased intrinsic activity propagation from mPFC in CM patients relative to EM patients may indicate the compensatory mechanism for attenuation of pain in patients with CM.

The correlation analyses revealed that the temporal lag values in the HP/PHG and mPFC were negatively associated with headache frequency across all patients. This means that migraine patients with a greater number of headache days per month displayed stronger intrinsic activity propagation from these two brain regions. In agreement with this finding, clinical features of migraines, including headache frequency, have been reported to be significantly correlated with structural or functional brain abnormalities of the HP/PHG [29,36] and mPFC [29,37,38]. Interestingly, the HP/PHG and mPFC are also key components of the DMN, a resting-state network important for cognitive processes [39] and pain processing [40]. Multiple lines of evidence have indicated that nodes of the DMN were major hubs of intrinsically propagated brain activity and were more susceptible under pathological conditions [15,41], such as post-traumatic stress disorder [19]. Collectively, our findings regarding these two regions suggested that propagation of intrinsic activity was disturbed in DMN during migraines and the extent of disruption may be linked to migraine chronification.

Moreover, earlier intrinsic activity was observed in the left STG/MTG and right PoCG/PreCG relative to the rest of the brain in both the CM and EM groups, when compared with the HC group. The PoCG/PreCG is implicated in the ascending trigeminal somatosensory pathway [42]. Therefore, increased intrinsic activity propagation from the PoCG/PreCG may be interpreted as impaired sensory-discriminative processing of pain in migraine patients. The STG/MTG belongs to the associative cortex and is important for auditory perception, multisensory integration, and language recognition [43,44,45]. It has also been reported to be part of the salience network, which is essential for the detection and directing of attention to salient stimuli [46]. It was possible that the identified temporal latency alteration of the STG/MTG may be related to sound hypersensitivity and disrupted salience response in patients with migraine. Our findings were partially supported by recent resting-state functional MRI studies that show aberrant effective connectivity of the STG/MTG [47] and sensorimotor cortex [48] in migraine patients. Furthermore, the absence of significant differences in STG/MTG and PoCG/PreCG lags between the two migraine groups indicated that atypical propagation of intrinsic activity in these two sensory processing-related brain regions were common features of individuals suffering from EM and CM.

Several limitations should be noted when interpreting the results of the current study. First, the number of participants for each group, especially for the CM group, was relatively small, so the acquired results should be considered as preliminary. Second, the HC and patient groups were not well matched in terms of age and gender distribution. However, it was unlikely that these factors greatly impacted our results, as the prominent findings showed no apparent changes after including the two variables as nuisance covariates. Importantly, temporal lag alterations in several regions were found to be correlated with clinical indices of migraine. Third, the lack of assessment of patients’ life quality and neuropsychological status made it impossible to comprehensively evaluate the impact of the migraine. Finally, for the cross-sectional study design, we could not ascertain whether the detected temporal latency abnormality was a consequence of recurrent migraine attacks or cause of this disease.

## 5. Conclusions

To conclude, the present study provided novel insights into the neural basis of migraines by examining the temporal lag structure of intrinsic brain activity. Migraine patients demonstrated altered intrinsic activity propagation in regions responsible for sensory perception, cognitive and affective processing of pain, including the sensorimotor cortex, lateral temporal cortex, mPFC and hippocampal complex. Earlier intrinsic activity in the left STG/MTG and right PoCG/PreCG seems to be a common manifestation for patients with EM and CM. Furthermore, the temporal lag values of intrinsic activity in the mPFC and HP/PHG may be potential brain imaging markers for assessing headache frequency that reflects the disease burden of migraines.

## Figures and Tables

**Table 1:** Demographic and clinical characteristics of patients and healthy controls.

	Male/Female	Age (Year)	Education (Year)	Days with Headache ^a^	VAS ^b^
EM (*n* = 39)	9/30	39.74 (11.59)	10.33 (4.02)	3.75 (2.64)	6.22 (1.77)
CM (*n* = 17)	9/8	49.59 (14.64)	9.71 (3.94)	19.56 (4.17)	7.24 (1.89)
HC (*n* = 35)	15/20	34.91 (10.89)	11.80 (4.92)		
*p* value	0.060 ^c^	<0.001 ^d^	0.195 ^d^	<0.001 ^e^	0.635 ^e^

^a^ Mean number of days with headache per month; ^b^ grading pain severity on a scale of 1 to10; ^c^ *p* value obtained with chi-square test; ^d^ *p* value obtained with one-way analysis of variance; ^e^ *p* value obtained with independent *t* test; continuous variables are given as mean (standard deviation). EM, episodic migraine group; CM, the chronic migraine group; HC, the healthy control group; VAS, visual analog scale.

**Table 2:** Brain regions showing significant temporal lag differences among groups.

Brain Region	Hemi	Voxel	MNI Coordinate (x, y, z)	Temporal Lag Value			Peak F Score
				CM	EM	HC	
STG/MTG	L	47	−48, −27, −3	−0.105	−0.118	0.129	7.59
PoCG/PreCG	R	64	60, −3, 15	−0.537	−0.387	−0.199	7.12
HP/PHG	R	47	24, −15, −21	−0.262	−0.050	0.135	8.96
HP/PHG	L	92	−24, −9, −21	−0.316	0.035	0.128	10.95
mPFC	L/R	80	−6, 45, −15	−0.172	0.152	−0.007	8.22

Hemi, hemisphere; MNI, Montreal Neurological Institute; L, left; R, right; CM, the chronic migraine group; EM, the episodic migraine group; HC, the healthy control group; STG, superior temporal gyrus; MTG, middle temporal gyrus; PoCG, postcentral gyrus; PreCG, precentral gyrus; HP, hippocampus; PHG, parahippocampal gyrus; mPFC, medial prefrontal cortex.

**Table 3:** Brain regions showing significant temporal lag differences between each pair of groups.

Brain Region	Hemi	Voxel	MNI Coordinate (x, y, z)	Peak T Score
EM VS. HC				
STG/MTG	L	32	−48, −27, −3	−3.84
PoCG/PreCG	R	48	51, −9, 15	−3.41
CM VS. HC				
STG/MTG	L	40	−48, −21, −15	−3.37
PoCG/PreCG	R	48	66, 3, 15	−3.57
HP/PHG	L	92	−24, −9, −21	−4.68
HP/PHG	R	47	24, −15, −21	−4.23
CM VS. EM				
HP/PHG	L	62	−18, −9, −21	−3.66
mPFC	L/R	80	6, 45, −15	−3.83

Hemi, hemisphere; MNI, Montreal Neurological Institute; L, left; R, right; CM, the chronic migraine group; EM, the episodic migraine group; HC, the healthy control group; STG, superior temporal gyrus; MTG, middle temporal gyrus; PoCG, postcentral gyrus; PreCG, precentral gyrus; HP, hippocampus; PHG, parahippocampal gyrus; mPFC, medial prefrontal cortex.

## Data Availability

The datasets used during the current study are available from the corresponding author upon reasonable request.
